# ECG-Based Detection of Early Myocardial Ischemia in a Computational Model: Impact of Additional Electrodes, Optimal Placement, and a New Feature for ST Deviation

**DOI:** 10.1155/2015/530352

**Published:** 2015-10-26

**Authors:** Axel Loewe, Walther H. W. Schulze, Yuan Jiang, Mathias Wilhelms, Armin Luik, Olaf Dössel, Gunnar Seemann

**Affiliations:** ^1^Karlsruhe Institute of Technology (KIT), Institute of Biomedical Engineering, Kaiserstraße 12, 76128 Karlsruhe, Germany; ^2^Siemens Healthcare Malaysia, Petaling Jaya, 46350 Selangor, Malaysia; ^3^Städtisches Klinikum Karlsruhe, Medizinische Klinik IV, Moltkestraße 90, 76133 Karlsruhe, Germany

## Abstract

In case of chest pain, immediate diagnosis of myocardial ischemia is required to respond with an appropriate treatment. The diagnostic capability of the electrocardiogram (ECG), however, is strongly limited for ischemic events that do not lead to ST elevation. This computational study investigates the potential of different electrode setups in detecting early ischemia at 10 minutes after onset: standard 3-channel and 12-lead ECG as well as body surface potential maps (BSPMs). Further, it was assessed if an additional ECG electrode with optimized position or the right-sided Wilson leads can improve sensitivity of the standard 12-lead ECG. To this end, a simulation study was performed for 765 different locations and sizes of ischemia in the left ventricle. Improvements by adding a single, subject specifically optimized electrode were similar to those of the BSPM: 2–11% increased detection rate depending on the desired specificity. Adding right-sided Wilson leads had negligible effect. Absence of ST deviation could not be related to specific locations of the ischemic region or its transmurality. As alternative to the ST time integral as a feature of ST deviation, the K point deviation was introduced: the baseline deviation at the minimum of the ST-segment envelope signal, which increased 12-lead detection rate by 7% for a reasonable threshold.

## 1. Introduction

The detection of ST deviations in patient electrocardiograms is an essential method for the diagnosis of myocardial ischemia. Both clinical studies [1–6] and simulations [7, 8] have shown that some forms of ischemia are visible neither in the standard ECG (see Table 1; sensitivity varies between 45% and 73% for detection of ST segment elevation) nor in body surface potential maps (BSPMs) [2, 4–6]. Such “electrically silent” ischemia results in non-ST elevation myocardial infarction (NSTEMI). Some studies suggest that sensitivity can reach 80% or even 100% (see Table 1) leveraging regional-specific ST elevation thresholds or computer-generated features including the QRS complex and the T wave. However, application of electrodes for BSPM comes at logistical cost; that is, it is usually not applicable for acute diagnosis. Muscle-brain (MB) type creatine kinase [9] and troponin tests [10] would be a gold standard but come at the expense of a time delay of several hours [4].

The intention of this work is to test the hypothesis that single or few additional ECG leads improve the sensitivity of the 12-lead ECG regarding the detection of early myocardial ischemia. While 80-channel BSPM naturally has a better ability to detect signal deviations, it is not well known where to place single additional electrodes in an emergency situation. Additional right precordial Wilson leads may improve sensitivity, yet with limited studies on the quality of evidence [11]. Kornreich et al. proposed an optimal placement for six electrodes [12], which differs completely from the standard precordial leads.

In this study, we start from the 3-lead and the 12-lead ECG and investigate the increase in detection rate when adding the right-sided Wilson leads compared to that of adding an optimally positioned electrode at any one in roughly 600 potential positions on the body. That is, we study what detection rates could ideally be achieved with an optimally placed electrode.

Detection rates are compared to those of a complete BSPM. It is also assessed if the magnitude of ST deviation and therefore the noise level in the signals play a role in whether additional leads result in improvements. Further, detection rates for different sizes, transmural extents, and locations of ischemia are presented.

The motivation of this work to be performed* in silico* arises from the difficulties in a clinical setting: 600-lead BSPM systems are not available and would be hard to apply for cases of acute ischemia. Moreover, ischemia locations and sizes cannot be characterized precisely, even with gadolinium delayed enhancement MRI. Computational modeling on the other hand facilitates a clear analytical approach here, producing statistics for different AHA segments and ischemia radii. Probably most important, ECGs of myocardial ischemia as early as 10 minutes after onset are typically not available in the clinical setting but will become more important in the coming years thanks to the advent of portable monitoring devices.

Large-scale simulation of ischemic events has previously not been feasible for a number of setups as high as in this study (765 constellations) as reaction-diffusion simulations take several hours for a single beat [7]. As in [13], to facilitate this work, a cellular automaton has been parameterized using the ischemia-adapted ten Tusscher model [14] and the monodomain solver* acCELLerate* [15]. Compared to other works from our group and others on electrode optimization [16–18] that used amplitude-based infarction models, the presented approach models acute myocardial ischemia and considers heterogeneities in resting membrane voltage (RMV), action potential (AP) amplitude, AP duration, and conduction velocity (CV) [13].

A variety of features in the ECG may be used to detect myocardial ischemia. Whether features other than ST deviation have negligible impact on 12-lead ECG sensitivity [19] or whether they improve sensitivity while reducing specificity [5] is under discussion. Nevertheless, the most prominent effects of myocardial ischemia are seen in the ST segment, and, in most emergency cases, assessment of ST deviation will dominate the diagnosis, as it does in most of the studies. Guidelines recommend an assessment of ST elevation at the J point [20]. However, it is in many cases very difficult to detect the J point reliably. The ST segment is therefore often localized at a certain number of milliseconds after the easy-to-detect R peak [21]. For this reason, we designed our analysis for a very simple feature, the ST deviation measured during 25 samples (*≙*48 ms) after the QRS. Motivated by the difficulty of detecting the J point and then assured by its superior performance over the aforementioned 48 ms ST deviation, we propose a new feature for ECG analysis, which we call the* K point deviation* (KPD): the baseline deviation at the minimum of the ST-segment envelope signal.

## 2. Materials and Methods

### 2.1. Electrophysiological Simulations

Electrophysiological simulations were carried out on three different datasets. We aimed at minimizing the impact of interindividual differences by spanning a large variability. While the anatomical model for subject VM was obtained by segmenting the Visible Man dataset, two models were produced by segmentation of MR images. Resolution was 2.27 × 2.27 × 4 mm^3^ (heart), 4 × 4 × 4 mm^3^ (thorax) for subject K (male; age: 61; posterior and posterolateral infarctions) and 1 × 1 × 1 mm^3^ (heart), 1 × 1 × 2 mm^3^ (thorax) for subject D (male; age: 27; healthy). The two models D and K comprised distinct tissue classes for the ventricles, skeletal muscle, fat, blood, lungs, kidneys, liver, and spleen (with only subject D having an atrial model and only subject K having a stomach model), whereas VM included 31 different tissue classes. However, the imaging data for the Visible Man dataset was not taken* in vivo*.

Fiber orientation was introduced for the ventricular myocardium using a rule-based approach [22]. For simulations of cardiac source signals, the anatomical datasets of the heart were interpolated to an isotropic voxel size of 0.4 mm (subjects VM and D) or 0.5 mm (subject K).

A cellular automaton (CA) was utilized to carry out ischemia simulations. Each voxel of the left and right ventricle can be activated by either adjacent voxels or an external stimulus. Once a voxel is excited, its transmembrane voltage (TMV) follows a predefined course, the AP. The TMV courses for physiological, nonischemic voxels were extracted from monodomain simulations using the reaction-diffusion system* acCELLerate* [15, 23] and the ten Tusscher electrophysiological model [24]. The approach described in [13] provided for transmural heterogeneity as well as differences in the AP duration at 90% repolarization (APD_90_) for each ventricular voxel. Anisotropic tissue conductivities inside the ventricular walls as well as the endocardial stimulation system were set according to [25].

The level of ischemia for each individual voxel was considered by assigning a so-called zone factor (ZF). A ZF of 0 represented healthy tissue, whereas voxels within the central ischemic zone (CIZ) were assigned a ZF of 1. Values in between represented voxels within the border zone (BZ). The effect of myocardial ischemia was modeled by modulating the TMV courses for the CA regarding four electrophysiological parameters: RMV, AP amplitude, APD_90_, and CV. The course of these parameters as a function of the ZF and the position within the ventricular wall was determined by conducting* acCELLerate* simulations as described in [13]. To this end, Weiss's extensions [7, 14, 26] to the ten Tusscher cell model that account for the three main biochemical effects of ischemia (extracellular hyperkalemia, acidosis, and hypoxia) were utilized. The model represents ischemic conditions at 10 minutes after onset (phase Ia, stage 2).

The left ventricular wall of all three datasets was divided using the 17-segment AHA scheme [27]. To run the study, hemispheric ischemic regions were placed at the center of each AHA segment. Their radii, comprising both the CIZ and the BZ, varied from 5 mm to 25 mm for two of the anatomical models. The upper limit for the Visible Man dataset was set to 30 mm due to the thicker ventricular wall. The center of the hemispheres was located at the endocardial surface of the myocardium as subendocardial myocytes need more oxygen due to stronger contraction and are therefore perfused more intensely [28]. Owing to this circumstance and because of the greater distance to the coronary arteries, electrophysiological changes can be first observed in the subendocardial layer after the onset of ischemia [29]. This situation is called subendocardial ischemia. If the occlusion lingers, ischemia spreads transmurally towards the subepicardium [30]. As soon as the entire wall is affected, the term transmural ischemia is used.

As the values for the extent of the BZ that can be found in the literature vary significantly [31, 32], three series of simulations were carried out using different BZ radii: 2.8 mm, 4.8 mm, and 9.6 mm. The time increment for the CA simulations was set to 0.1 ms and the results were saved every 2 ms.

TMVs obtained through the CA simulations were interpolated on tetrahedral meshes in order to carry out electrical field finite element calculations. Extracellular potentials on the body surface of the respective anatomies (BSPMs) were computed using the bidomain model as in [25]. The conductivity tensors *σ*_*e*_ and *σ*_*i*_ were set according to [33]. From these BSPMs, electrode signals were extracted at 593–815 equally distributed positions on each torso with an average distance of 4 cm between electrodes.

To validate the ischemia implementation for the CA, 20 ischemic ECGs were compared to reference simulations carried out using the full monodomain solver* acCELLerate*. The root mean square error in the ST segment (ranging from 120 ms to 250 ms) was smaller than 0.02 mV for more than 50% of the 180 computed lead signals. Aberrations larger than 0.2 mV were observed in 12 signals. The reason for these deviations was broadened QRS complexes ranging beyond 120 ms due to ischemia effects.

### 2.2. Features of ST Elevation

The ST segment deviation (STSD) for a specific channel was defined as the absolute value of the mean of the 25 samples between 110 ms and 158 ms:

(1) $$\begin{array}{c} \text{STSD}=\underset{e}{\max}\left(\frac{1}{25}\left|\overset{25}{\underset{t=1}{\sum }}b\left(e,t\right)\right|\right), \end{array}$$

where *b* is the ECG signal, *e* is the electrode channel, and *t* is the time step.

**Figure 1:**
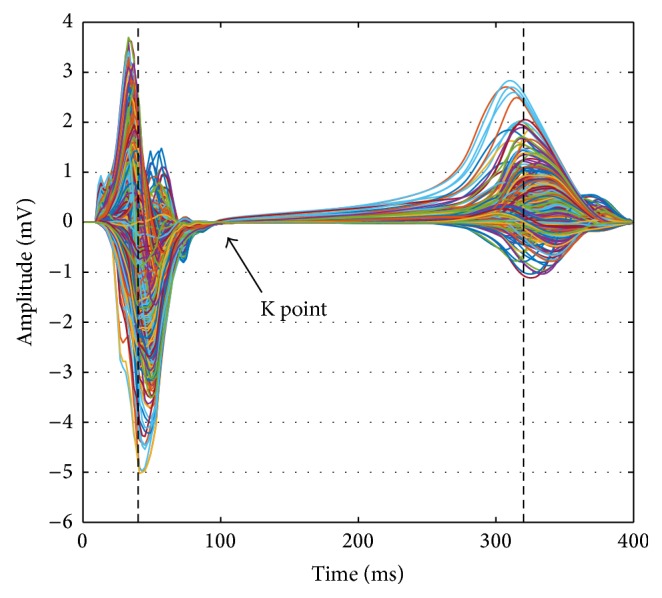
593 electrode signals of the physiological simulation for subject D. The arrow points to the K point being defined as the time step between 40 ms (R peak) and 320 ms (T peak) (dashed lines) for which the maximum absolute value over all observed leads is minimal.

As pointed out in the introduction, it is challenging to detect the ST segment and especially the J point in ECG analysis. Also, the previously defined ST deviation is sensitive towards interference with the QRS or T wave in case of even slight errors in the timing of annotations. We therefore propose the definition of a *K point* as substitute for the J point. For a given set of lead signals, the lead with the maximum absolute value is determined individually for each time step between the R peak (40 ms) and the T peak (320 ms) (envelope of absolute values). The K point is then defined as the time step for which this envelope signal is minimal (see Figure 1). The corresponding K point deviation (KPD) is thus

(2) $$\begin{array}{c} \text{KPD}=\underset{t}{\min}\left(\underset{e}{\max}\left|b\left(e,t\right)\right|\right). \end{array}$$

Using this definition, alternating ST deviation (e.g., elevation at the beginning of the ST segment followed by depression) can still be detected as opposed to the STSD.

### 2.3. Study Setup and Lead Systems

This work investigates the limitations of different electrode setups in representing ST deviations caused by myocardial ischemia. To this end, standard 3-channel ECGs (I, II, III, aVR, aVL, and aVF), 12-lead ECGs, and body surface potential maps (BSPMs) of 593–815 electrodes were acquired from the thoraces of the simulation datasets. All unipolar leads and the BSPMs were measured against Wilson central terminal (WCT).

It was then assessed if additional ECG leads can improve sensitivity of the 3-channel or 12-lead ECG. The improvement by adding the four right-sided Wilson leads *V*_3*R*_ through *V*_6*R*_ (“12 + *R*”) was investigated. Moreover, we looked at all the 593–815 electrodes with respect to WCT “BSPM”. Considering the whole BSPM improved detection rates significantly when looking at KPD, the optimum position of a 10th electrode was determined considering all ischemic setups for a given subject and threshold “12 + 1”. This scenario will be more feasible than BSPM during emergencies; however, it assumes that the location for this additional electrode is known for a specific subject. We investigated if such an optimal position can exist, that is, if it is consistent across subjects by mapping the individually optimal positions to the other 2 thoraces. The 12 + 1 scenario was not considered for the STSD as the improvement by looking at the whole BSPM as compared to the 12-lead ECG was already limited; thus, the benefit of just one additional electrode was negligible.

## 3. Results

### 3.1. Acute Diagnosis Using ST Deviation

**Figure 2:**
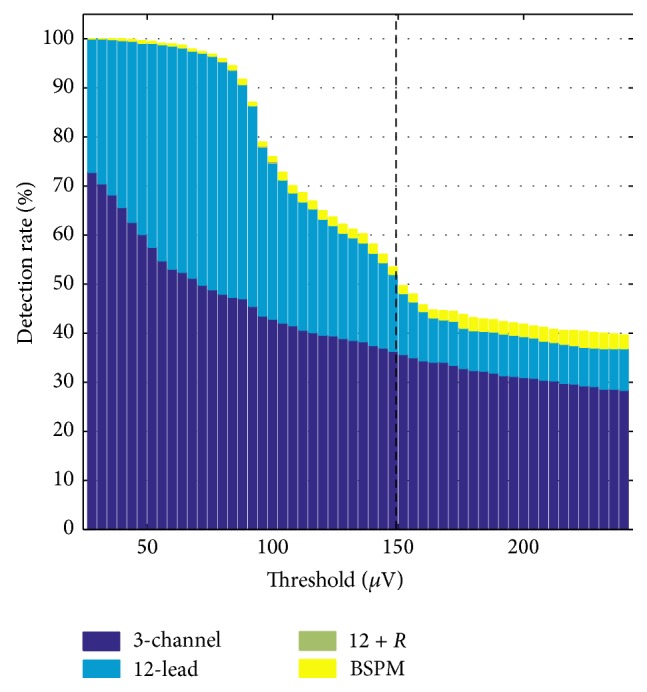
Ischemia detection rates based on STSD for different thresholds and ECG lead systems. The dashed line shows the largest STSD seen in physiological simulations without any ischemic tissue considering all electrodes. The improvement by considering the right-sided Wilson leads “12 + *R*” was limited and can hardly be seen in the stacks.

ST segment deviation in the physiological ECGs without any ischemic tissue was nonzero and amounted to 96/99/149 *μ*V for VM/K/D considering all electrodes (dashed line at 149 *μ*V in Figure 2). The electrode showing the highest STSD with respect to WCT in the physiological simulation was the one being used to compute lead *V*_2_ for subjects VM/K and *V*_3_ for subject D. Thus, the highest physiological deviation was the same in all scenarios including at least the 12-lead electrodes.

Figure 2 shows the detection rates obtained for certain STSD thresholds when looking at different sets of lead signals spanning all subjects. The threshold defines the minimum STSD according to 1 which needs to be observed for a given set of leads to classify an ECG as ischemic. Compared to 3-channel ECG, the 12-lead ECG improved the detection rate by between 48% (threshold = 76 *μ*V) and 8% (threshold = 240 *μ*V). Further improvement by considering the right-sided Wilson leads was limited to a maximum of 3 ischemic setups (*≙*0.4%). Leveraging STSDs from the whole BSPM improved the detection rate by a maximum of 3.3% and less than 2% for thresholds <176 *μ*V.

To facilitate statistics that are independent of the threshold, detection rates were averaged for 54 threshold steps equidistantly distributed between 24 *μ*V and 240 *μ*V in the following. This approach was chosen as the question of finding the optimum threshold balancing sensitivity and specificity cannot be answered by this study.

Looking at different sizes of the ischemic regions, detection rates increased with increasing ischemia size ranging from 41% for a total radius *r* = 5 mm to 95% for *r* = 30 mm when looking at the 12-lead ECG. The improvement by considering STSDs in all BSPM leads was limited; however, it was the biggest for medium-sized ischemic regions (0%/0.9%/2.81%/2.99%/0.94%/0.39% improvement of detection rate for *r* = 5/10/15/20/25/30 mm).

In general, subendocardial ischemia was harder to detect than transmural ischemia. Detection rates for transmural setups were higher by 1.6%–10.2% for small to medium-sized ischemic setups (*r* = 5–15 mm) and better by 20.8% for *r* = 20 mm. The case of 25 mm radius was an exception: here, the detection rate was better by 8.2% for subendocardial setups of which all were detected. Findings for transmural ischemia are not biased by the volume of the ischemic region. The fraction of ischemic voxels (CIZ + BZ) in the left ventricle was very similar for transmural and subendocardial ischemic setups of a specific radius (e.g., 3.7% for transmural ischemia versus 4.1% for subendocardial ischemia for *r* = 15 mm and 8.2%/10.1% for *r* = 20 mm).

Different BZ sizes had only little effect on detection rates. However, the smaller the BZ, the easier the detection of ischemia. Rates for small/medium/large BZs in the BSPM scenario were 68.4%/65.0%/63.4%. Looking at the 3 different subject models, detection was the easiest for subject D, followed by K and VM in all lead systems. Rates for VM, K, and D were 33.2%/52.9%/53.5%, 46.4%/64.4%/67.2%, and 46.4%/77.8%/79.2% for 3-channel/12-lead/BSPM.

### 3.2. K Point Deviation for Acute Diagnosis

**Figure 3:**
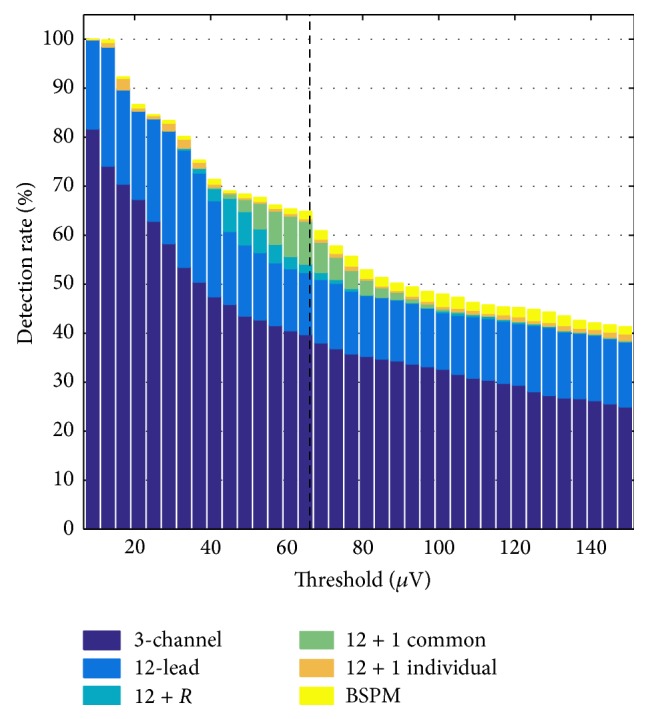
Ischemia detection rates based on KPD for different thresholds and ECG lead systems. The dashed line represents the largest KPD observed in physiological simulations without any ischemic tissue considering all electrodes.

The KPD observed in ECGs obtained from physiological reference simulations without any ischemic tissue ranged from 3.4 *μ*V for subject D in the 3-lead system to 66 *μ*V for subject K in the BSPM lead system (dashed line in Figure 3). Details are shown in Table 2. Regarding the ischemic ECGs, the temporal position of the K point showed variations, which were more pronounced for scenarios covering fewer leads (mean ± standard deviation 83 ± 139/76 ± 132/71 ± 126 ms for 3-channel/12-lead/BSPM). For small ischemia radii, the position did not differ significantly from the one in the physiological signal. For setups with *r* ≥ 10 mm, the K point moved towards the T wave in most cases. Sometimes, the K point was located within the QRS complex due to massive ST segment deviations in some leads especially in the 12 + 1 and BSPM scenarios. For *r* = 5/10/15/20/25/30 mm, the temporal position of the K point was 27 ± 3/27 ± 3/32 ± 16/53 ± 40/78 ± 49/80 ± 43 ms in the 12-lead scenario.

**Figure 4:**
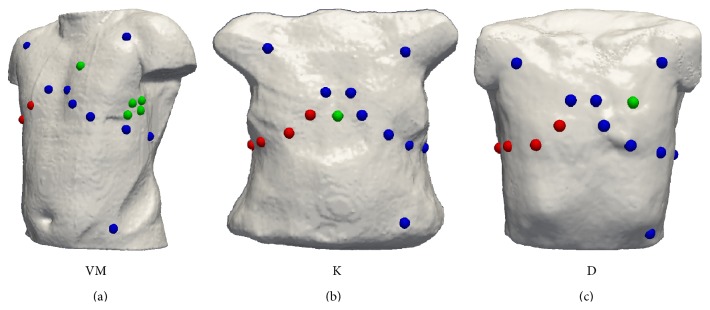
Optimum positions for 10th electrode considering KPD (green spheres) together with 12-lead electrodes (blue spheres) and right-sided Wilson leads (red spheres). For VM, all 5 indicated locations yielded the same detection rate. The position yielding the best combined detection rate for all subjects was the same as found for D individually.

Figure 3 shows the KPD-based detection rates for the different lead systems. The extension from 3 channels to 12 leads accounted for an improvement of around 15% which was almost constant for all threshold values. Adding further leads brought about most improvement for threshold values between 40 *μ*V and 70 *μ*V. In this region, a detection rate of around 65% could almost be sustained by adding an additional electrode when raising the threshold. Adding right-sided Wilson leads helped to detect ischemia mainly for thresholds between 40 *μ*V and 50 *μ*V. However, the gain in detection rate by adding these four additional electrodes was always less compared to adding one additional electrode whose position had been optimized for a given subject. Please note that this electrode position was the same for all ischemia radii, locations, and BZ extents. The additional improvement by looking at the remaining several hundred electrodes of the BSPM was limited to less than 2.5%. It was most beneficial for higher threshold values, though. Using a subject-specific location for the 10th electrode (Figure 3: 12 + 1 individual) yielded an average increase in detection rates by 0.8% compared to one common location for all 3 subject models (12 + 1 common).  Figure 4 shows the position of the individually best electrode locations and the best common position.

As the aim of this work is to evaluate the sensitivity capability of different lead systems, the presented results focus on KPD. In brief, Figure S1 in the Supplementary Material available online at http://dx.doi.org/10.1155/2015/530352 indicates that basing ischemia detection on pure K point elevation reduces detection rates in all electrode scenarios. However, additional electrodes can compensate for the disproportionate decrease in the 12-lead scenario (−4.4%/−8.9%/−5.2% for 3-channel/12-lead/BSPM at the greatest physiological threshold).

**Figure 5:**
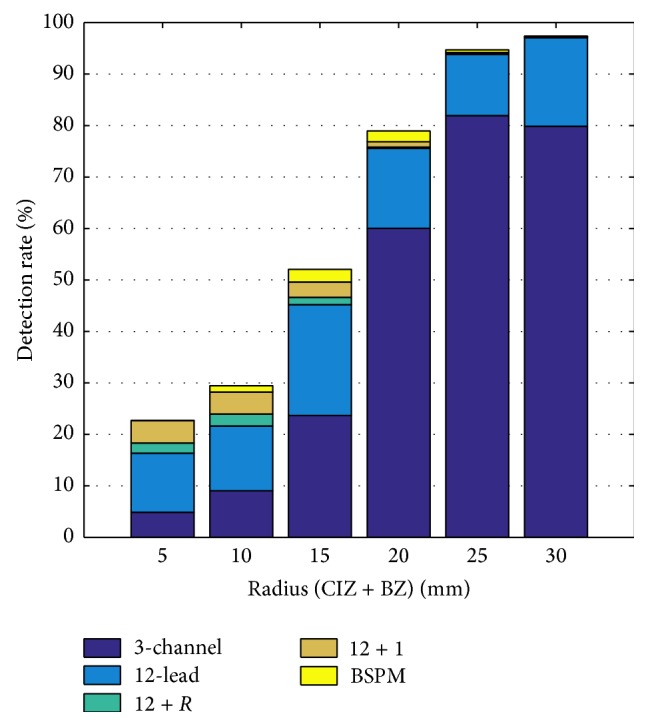
Ischemia detection rates based on KPD for different ECG lead systems and different total radii of the ischemic region averaged over the aforementioned set of thresholds.

Below, detection rate statistics were again averaged over 36 threshold steps linearly spaced between 9 *μ*V and 150 *μ*V in order to minimize the uncertainty associated with threshold selection.  Figure 5 shows that adding further electrodes to the 12-lead ECG achieved higher detection rates mostly for small and medium ischemia radii. Larger ischemic regions were easier to detect than smaller ones using KPD as well.

Compared to subendocardial setups, detection rates for transmural setups were higher by 3.2% for small ischemic regions (*r* = 5 mm) and better by 28.9% for medium-sized ischemic regions, which almost doubled the detection rate in that case. For *r* = 25 mm, detection rate was better by 5.9% for subendocardial setups of which all were detected as was the case for STSD.

The effect of the BZ size was, again, small. Average detection rates were 59.1%/54.9%/53.2% for small/medium/large BZs. The BZ dependencies of additional electrode related improvements were insignificant.

**Figure 6:**
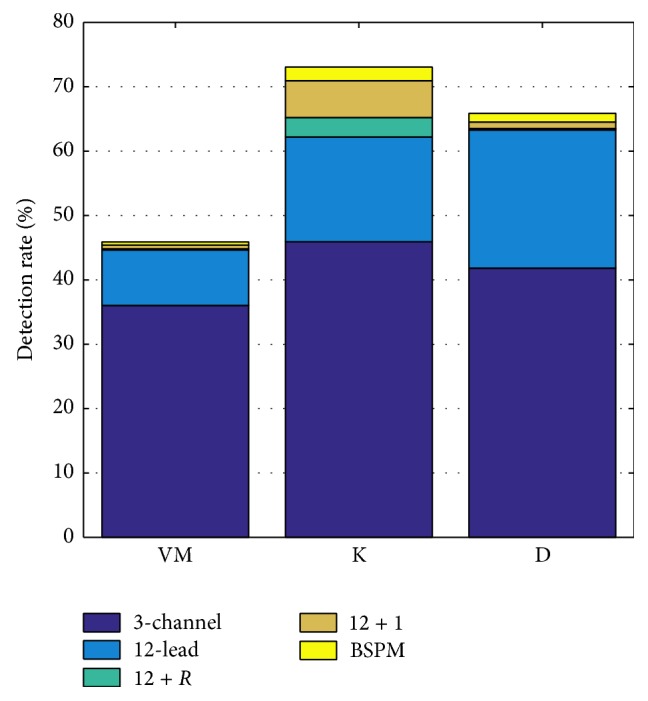
Ischemia detection rates based on KPD for different ECG lead systems and different subjects averaged over the aforementioned set of thresholds.

Considering the different subjects, ischemia was the hardest to detect in VM. However, using KPD, it was easier to detect in K than in D (see Figure 6) as opposed to using STSD where it was the other way round. Adding additional electrodes increased detection rates the most in subject K. The 12 + 1 scenario yielded a detection rate increase of 0.7%/8.8%/1.2% for subjects VM/K/D compared to the 12-lead ECG.

**Figure 7:**
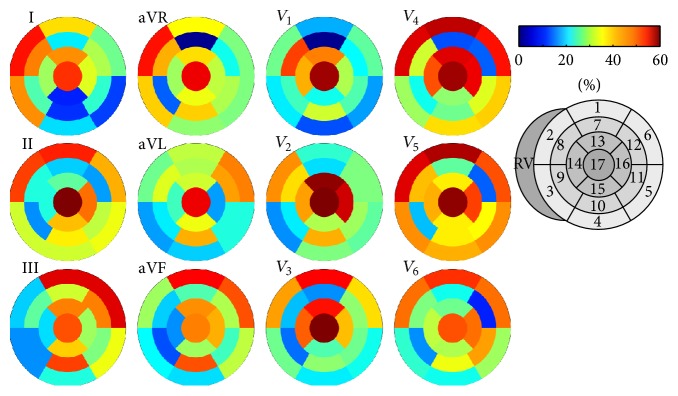
Ischemia detection rates based on KPD for single 12-lead channels per AHA segment averaged over the aforementioned set of thresholds. The temporal position of the K point was determined using all 12 leads.

Figure 7 shows the average detection rates considering the KPD in only a single lead at a time. The temporal position of the K point was determined using all 12 leads for this analysis. For Figure S2 in the supplementary material, only K point elevation was considered. The sensitivity focus pivoted towards lateral AHA segments in the midapical layers for more lateral Wilson leads (*V*_4_ through *V*_6_) for pure K point elevation. This elevation analysis furthermore revealed the importance of the limb leads III, aVR, and aVL for the detection of basal ischemia. When also considering K point depression (Figure 7), additional sensitivity in opposite segments, particularly in the midbasal layers, resulted in a less distinct pattern (compare, e.g., *V*_6_). While apical detection rate was high in all leads when considering KPD, it was significantly above average only for the contiguous limb leads II, aVF, and III, as well as *V*_1_ and *V*_5_ when only considering K point elevation.

**Figure 8:**
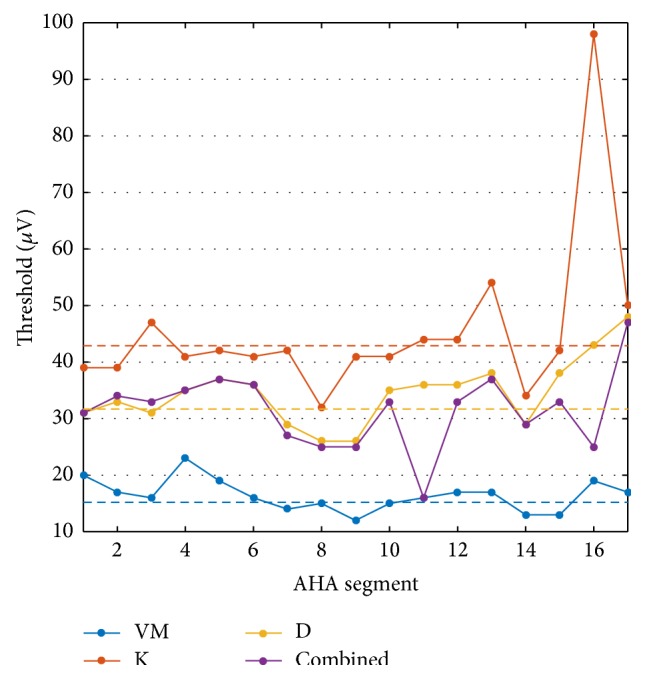
Threshold to detect at least 80% of all ischemic setups in a specific AHA segment based on KPD in the 12-lead scenario. Besides subject-individual curves, the overall threshold to detect at least 80% in the whole 3-subject population (combined) is shown. Dashed lines represent the largest physiological KPD observed in the individual subjects.

Additionally, the maximum threshold to detect ≥80% of all ischemic setups for a given subject in a specific AHA segment was determined to gain insight whether ischemia translates to smaller ST deviations in some regions than in others. Looking at the detection rates subdivided by AHA segment did not reveal a significant pattern considering absolute detection rates as well as improvements by introducing additional leads. The maximum threshold to detect at least 80% of all ischemic setups in a given segment is shown in Figure 8. Segments 8 and 14 were calling for lower thresholds, hence harder to detect, and segments 4, 16, and 17 were easier to detect than average. However, the curves for different subjects did not intersect, thus showing comparatively constant interindividual differences. Maximum thresholds to detect at least 80% of all ischemic events in a particular segment were greater than the greatest physiological deviation (dashed lines in Figure 8) more often than not when considering subjects individually. However, only segment 17 would meet the 80% requirement when choosing the greatest physiological KPD across all subjects as threshold.

## 4. Discussion

In this work, a comprehensive* in silico* study was performed to test the sensitivity of BSPMs against the 12-lead and the 3-channel ECG in detecting acute myocardial ischemia at 10 minutes after onset (phase Ia, stage 2). Also, the question was investigated whether right-sided Wilson leads or a single additional electrode would improve sensitivity for the 12-lead ECG, assuming the ideal case that its position can be known. It was then studied if such an ideal position can be found that is valid across subjects.

For the ST segment deviation, the standard 12-lead ECG could detect more ischemic setups (64.2% ± 24.9% for threshold values between 24 *μ*V and 240 *μ*V) than a 3-channel ECG (41.4% ± 11.8%) while right-sided Wilson leads in addition hardly improved detection rates at all. Our results regarding the right-sided Wilson leads underline the results in [34] and may explain why little clinical validation exists on their effect [11]. The supplementary information gained by covering STSDs on the whole torso increased detection rates by 2-3% but was not a game changer. Thus, the benefit of using BSPMs with respect to ST segment integrals was limited in our study compared to 12-lead ECG. This also implies that a single additional electrode with ideal placement could not possibly improve detection rates significantly. The improvements in sensitivity reported in the literature for BSPMs over the 12-lead ECG are, however, much larger: Ornato et al. [4] found a relevant additional sensitivity compared to the 12-lead ECG when using the BSPM (100% versus 72.7% for kinase MB and 92.9% versus 60.7% for troponin tests). Shifts were also larger in [2], where sensitivity improved from 45% for the 12-lead ECG to 64% for BSPMs, or in [5] where sensitivity increased from 45% to 92%. One potential reason for these differences is that, in contrast to clinical works, detection rates in this study were computed with respect to the possible variants of the studied ischemic events at an equal distribution, not with respect to their occurrence in the presenting patient cohort. More importantly, we were looking at early ischemia at 10 minutes after onset in this study which differs significantly from the time when clinical ECGs are typically acquired.

Larger ischemic regions were easier to detect (95%) for the 12-lead ECG than small ones (41%). On the one hand, this is not surprising due to their larger heart surface and thus signal. On the other hand, this finding seems to contradict the results by Wilhelms et al. [7, 26, 35] at first sight. In their studies, subendocardial ischemia caused ST depression, which changed into pronounced ST elevation for larger transmural ischemic regions. For intermediate sizes, ischemia became “electrically silent.” Our simulations based on the phenomenological ischemia model showed the same behavior when investigating a single ischemic event. A comparable scenario (AHA segment 4; BZ: 4.8 mm; subject D) yielded STSD of 94/33/123/264 *μ*V for *r* = 10/15/20/25 mm (*≙*1.2%/2.8%/5.3%/8.6% ischemic tissue). The reason for this transition not being visible in Figure 5 is that always a whole set of ischemic events is investigated. Due to inhomogeneous wall thickness, thus different minimum radii to gain transmurality, this effect of “intermediate silence” is buried under the influence of stronger signal deviations caused by larger ischemic regions.

The extent of the border zone had little impact on the detection rates, which suggests that this parameter may not need to be varied in future studies of ischemia simulations, despite its large span of literature reporting.

As alternative to the STSD, the KPD was proposed in this work as a favorable method to measure ST deviation. For comparison of these features, detection rates have to be evaluated for an appropriate threshold. As variations in the physiological ECG due to various effects are beyond the scope of this study, the threshold was naturally best set above the greatest STSD or KPD found in the three physiological, nonischemic simulations. Specificity turns from 0 here to 1 for greater thresholds. As there will be a smoother fading in a real patient population, a slightly greater threshold is assumed to correspond to a practically reasonable balance between sensitivity and specificity. The ST segment deviation and K point deviation features were therefore compared at 1 and 1.5 times their greatest physiological threshold.

The proposed KPD feature produced a detection rate of 52.4%/64.8% (12-lead/BSPM) compared to 52.0%/53.5% for the STSD feature at the greatest physiological threshold. At 1.5 times the threshold, detection rates were 44.6%/48.3% for the KPD and 37.5%/40.5% for the STSD. The KPD feature can therefore be assigned a better sensitivity for evaluation at the greatest physiological threshold when using BSPMs and a considerably better sensitivity for an evaluation at 1.5 times the physiological threshold also in the 12-lead scenario. This implies a gain in detection rates for BSPMs compared to the 12-lead ECG that is closer to the reported gains in [2, 4, 5]. For very low thresholds, the STSD feature delivered greater sensitivity than the KPD feature, yet without practical implications, as these thresholds cannot be used for diagnosis. Compared to the vector magnitude, which is proposed in [6] as the maximum minus the minimum signal at the J point, the KPD includes a definition on how to identify the time point of evaluation. While no significant changes in sensitivity or specificity were found in [6] for BSPMs when using the vector magnitude compared to using the ST elevation at the J point, the KPD deviation feature in the present study leads to improved detection rates.

Considering pure K point elevation instead of deviation comprising both elevation and depression decreased detection rates as expected (see Figures S1 and S2). Interestingly, the loss of sensitivity was particularly pronounced in the 12-lead scenario. The additional loss compared to the 3-channel scenario could be almost compensated by adding additional leads. This result can be explained by the fact that ischemia causing elevation in a particular lead translates to depression in opposite leads and advises against solely considering elevation, particularly in situation with limited lead coverage.

For the KPD as measure of ST deviation, the initial questions addressed in this work can be asked again. Detection rates for the 12-lead ECG were again considerably better than for the 3-lead ECG. Larger ischemia was easier to detect than small ischemia, yet with the surprising difference that detection rates for KPD were lower than those for STSD for radii ≤15 mm.

Compared to STSD, some significant qualitative differences were found, though. Just below the largest physiological deviation, detection rates gained significantly from adding right-sided Wilson leads, which is unfortunately of no use for diagnosis. For a single additional electrode with optimized position though, this gain can be appreciated, even if a common optimum position is chosen for all subjects. Here, just as the entire BSPM, the single electrode extension literally brought about the benefit of using the KPD feature, whereas it contributed less to higher detection rates of the feature when considering higher thresholds. Interestingly, the detection rate could be increased significantly stronger by additional electrodes for subject K compared to the other two subjects.

The optimum positions for a single additional electrode (Figure 4) were relatively close to the standard leads (*V*_1_/*V*_2_ for subject K, *V*_3_/*V*_4_ for subject D, and *V*_4_/*V*_5_ for positions in subject VM). This suggests that a denser spatial sampling in the area of the breast is more beneficial than additional electrodes on the back or to the right. The positions correspond approximately to the six optimum anterior positions found in [12, Figures 4–6] (2 for anterior ischemia, 2 for inferior ischemia, and 2 for posterior ischemia). The missing concordance regarding the posterior positions can be attributed to the different study designs. In [12], the ECGs were computed with respect to a second electrode on the patients' back, thus aiming at optimized pairs of two electrodes, whereas the present study optimized the position of a single additional electrode to the 12-lead ECG. Another study by Finlay et al. also found precordial leads to be best-suited for the detection of myocardial infarction in a 6-channel system [36].

NSTEMI did not differ in general from STEMI regarding their location in the heart. Our findings of minor differences between regions are surprising as they contradict suggestions that myocardial ischemia is particularly difficult to detect in the posterior segments (AHA segments 4, 10, and 15) [37]. A possible reason for this difference is the later phase of ischemia in [37]. Sensitivity of particular leads towards ischemia in specific regions of the left ventricle corresponded with earlier studies as reviewed in [38] when considering pure K point elevation (see Figure S2 in Supplementary Material). Comparing these results with KPD-based detection revealed that taking depression into account as well renders detection in opposite segments possible in many cases. Thus, depression is particularly important to consider in scenarios with limited electrode coverage of the thorax. In line with [37], improvements with BSPMs over the 12-lead ECG in [2] were mostly due to posterior or right-sided ischemia being detected. In our study, however, the septal segments 8 and 14 showed signs of being especially difficult to detect. Taking into account the absolute threshold values in Figure 8 with respect to the KPD seen in the physiological simulations (Table 2), the results of our analyses should not be considered as suggestions for KPD thresholds in clinical practice as they would translate to unacceptable specificity. The aim of this analysis is rather to point out the theoretical limit. In practice, the threshold would need to be traded off against sensitivity. Even in combination with further symptoms, sensitivity might thus be relatively low for ischemia as early as 10 minutes after onset.

For both ST deviation features, results for detection rates are at the lower end of the reported values in the literature. For the 12-lead ECG, average detection rates with STSD and KPD were 52.0% and 52.4%, which is within the lower end of the reported 45%–73% sensitivity range [1, 2, 4]. For BSPMs, detection rates were 53.5% and 64.8%, which is at the lowermost end of the reported results, which range from 64% to 100% [2, 4]. Hence, it must be concluded that ST deviations in our physiological reference simulations were relatively high and those of simulated ischemia were relatively low. This may well be attributed to the great portion of small and therefore early ischemic events that do not present in the clinic at the same proportion.

The largest STSD in ECGs from physiological reference simulations was observed for subject D, who is the youngest of our study population. This result corresponds to [39], where the prevalence of ST segment elevation seen in 12-lead ECGs of healthy men declined with increasing age. However, it has to be stressed that in our study only the geometrical model and not the electrophysiological model is subject-individual, thus age-dependent. In [39], criteria for ST segment elevation were met by 95% of young men in at least one of leads *V*_1_ through *V*_4_. Yet for KPD, the youngest subject D was somewhere in the middle between subjects VM and K; that is, the KPD feature has different characteristics compared to the STSD, which has to be taken into account for comparison with clinical literature.

### 4.1. Limitations

The present study is based on models and simulations which can only approximate real anatomy and physiology to a certain degree. Conclusions from the study are dedicated to serving a better understanding of the underlying effects.

While providing valuable insight into sensitivity characteristics of different lead systems and features of ST deviation, this study does not allow for an assessment of the specificity. False-positive rates cannot be given as variations of the ECG due to the fact that other causes than ischemia are beyond the scope of this work. Therefore, the threshold values used in this study are not to be considered as a suggestion for clinical practice. This should be kept in mind when interpreting the presented detection rates. Another aspect that should be stressed again is that the distribution of ischemia sizes and locations is uniform as opposed to patient cohorts in clinical studies. However, this allows for statistics regarding the coverage of the whole left ventricle.

Animal studies have demonstrated that during the evolution of myocardial ischemia the affected zones may have distributed geometric shapes [40], and they may obviously extend into multiple AHA segments. We decided to base this study on a rather simple model of isolated hemispherical ischemic zones which are typical for distal vessel occlusion allowing to unveil the characteristics regarding location, total, and border zone size without being obscured by other factors. Future work could take into account ischemic regions computed using coronary artery tree diffusion models which may also affect the right ventricle.

Moreover, the study may be extended to later phases of ischemia [7] aiming at clinical rather than preclinical diagnosis in a follow-up work.

## 5. Conclusion

In this work, we addressed the question which kind of early left ventricular ischemia can be detected using ECG features of different lead systems. The main goal was to unravel common characteristics of NSTEMI and to propose a better-suited feature to detect ST deviation. Moreover, we aimed to identify a good tradeoff between the cost of additional electrodes and the gain in sensitivity. By these means, we are confident to contribute to the improvement of the sensitivity of ECG-based diagnosis of acute myocardial ischemia. Towards this end, a computational study of the ECG changes due to left ventricular ischemia of various sizes, locations, and border zone extents was conducted for a virtual population of 3 subjects. Using an* in silico* approach allowed to investigate changes in the ECG due to well-defined changes of the properties of the ischemic region.

Regarding the different lead systems, around 20% additional ischemic events could be detected using the 12-lead ECG compared to 3-channel ECG. The right-sided Wilson leads did not improve sensitivity significantly for relevant thresholds. Covering the whole torso by analyzing a BSPM formed by several hundred electrodes improved detection rates by only 2-3% using the STSD, while it yielded an additional improvement of up to 12.5% using the newly proposed KPD feature. The latter was defined as the baseline deviation at the minimum of the ST-segment envelope signal. For the KPD feature, the improvement using just one additional electrode to the 12-lead ECG was almost as large as considering a complete BSPM even when optimizing its position across all 3 subjects. Altogether, the KPD resulted in a higher sensitivity compared to the STSD. These results highlight the importance of a good feature to detect ischemia induced ECG change. They also suggest focusing future research on such features using high spatial resolution regarding the precordial wall while considering both signal elevation and depression rather than covering the right side of the thorax or the back.

Looking at the characteristics of the ischemic regions, the BZ extent and transmurality did play a minor role and thus might not be needed to be investigated in detail in future studies. Larger ischemic regions were easier to detect than small ones and the location did not translate to a distinct sensitivity pattern. In fact, global interindividual differences outweighed intraindividual changes in ischemia location.

Our results add to the knowledge concerning ischemia induced ECG changes and our findings may aid in eventually reducing the share of NSTEMI in acute diagnosis, thus increasing the number of patients benefiting from immediate treatment.

## Supplementary Material

Figure S1. Ischemia detection rates based on K point elevation for different thresholds and ECG lead systems. The dashed line represents the largest K point elevation observed in physiological simulations without any ischemic tissue considering all electrodes.
Figure S2. Ischemia detection rates based on K point elevation for single 12-lead channels per AHA segment averaged over the aforementioned set of thresholds. The temporal position of the K point was determined using all 12 leads.

## Figures and Tables

**Table 1:** Clinical studies concerning the detection of acute myocardial ischemia. Ranges for [4] represent validation against troponin/creatine kinase MB.

Sensitivity	Specificity	Detection method
12-lead ECG		
45%	92%	Physician, ST elevation [1]
45%	92%	Physician, modified AHA/ESC guidelines [41], in [5]
45%	94%	Physician, ST elevation [2]
65.9%	86.3%	Artificial neural network, six ST-T features [3]
60.7–72.7%	96.4–97.1%	Physician, ST elevation [4]

80-channel BSPM		
64%	94%	Rule-based, QRST features [2]
76%	92%	Physician, computed QRST features [5]
80%	92%	Physician, computed ST features [6]
92.9–100%	94.9–96.5%	Physician, computed QRST features [4]

**Table 2:** Maximum KPD in *μ*V observed in different lead systems of ECGs from physiological reference simulations without any ischemic myocardium. The maximum deviation was observed in the lead given in parentheses where *o* stands for any nonstandard electrode in the BSPM case.

Subject	3-channel	12-lead	12 + *R *	BSPM
VM	7.6 (II)	15.2 (*V*_2_)	15.2 (*V*_2_)	15.5 (*o*)
K	26.3 (I)	42.9 (*V*_4_)	42.9 (*V*_4_)	66.1 (*o*)
D	3.4 (II)	31.7 (*V*_5_)	31.7 (*V*_5_)	31.7 (*V*_5_)
