# Supplementary figures and images for: ECG-Based Detection of Early Myocardial Ischemia in a Computational Model: Impact of Additional Electrodes, Optimal Placement, and a New Feature for ST Deviation
Source: Biomed Res Int. 2015 Oct 26;2015:530352. doi: 10.1155/2015/530352 (PMC4637443; doi:10.1155/2015/530352)

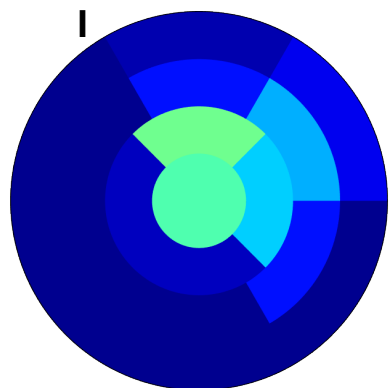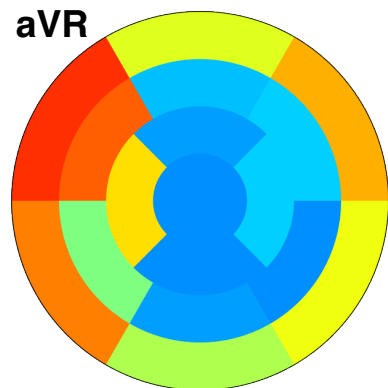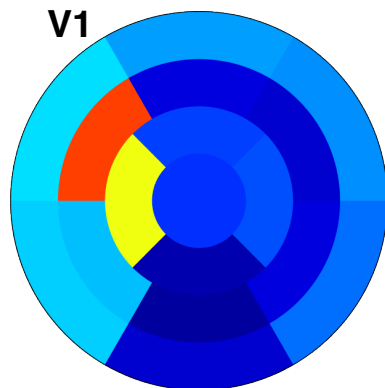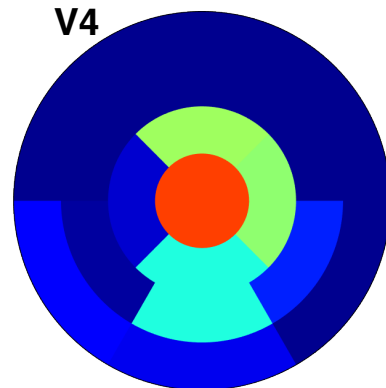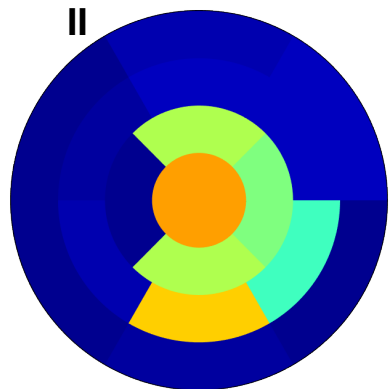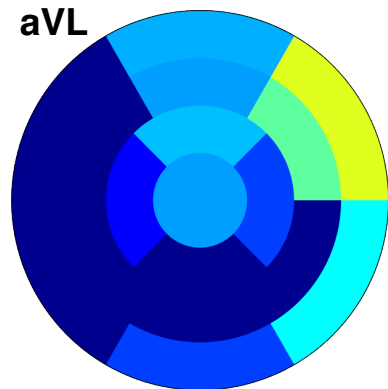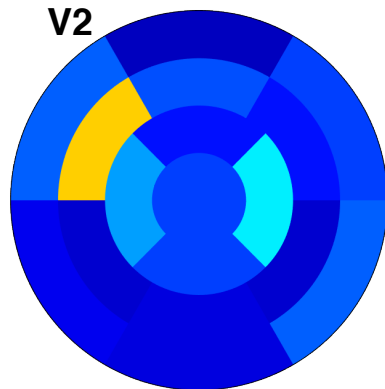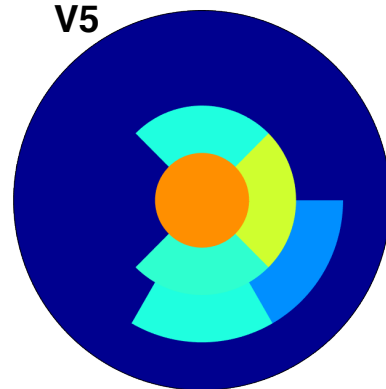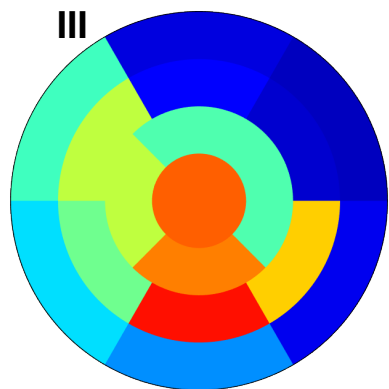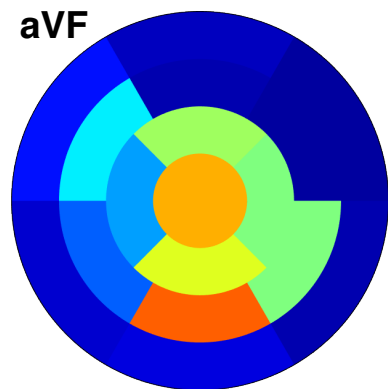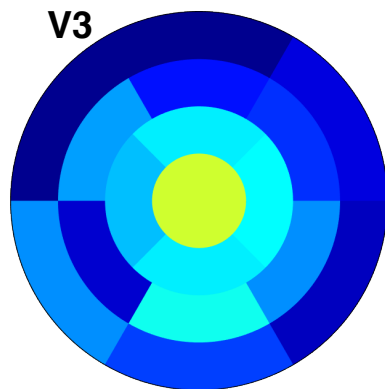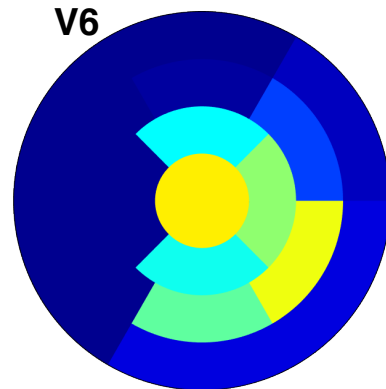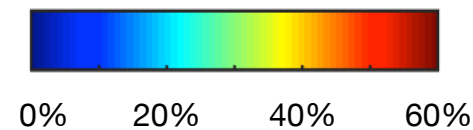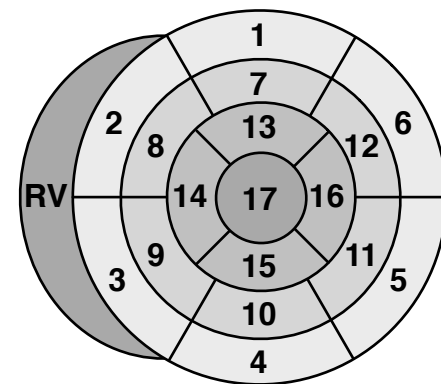

Supplement: Supplementary file 1 — Figure S1. Ischemia detection rates based on K point elevation for different thresholds and ECG lead systems. The dashed line represents the largest K point elevation observed in physiological simulations without any ischemic tissue considering all electrodes. Figure S2. Ischemia detection rates based on K point elevation for single 12-lead channels per AHA segment averaged over the aforementioned set of thresholds. The temporal position of the K point was determined using all 12 leads. [file 530352.f1.zip › leadsElev.pdf]

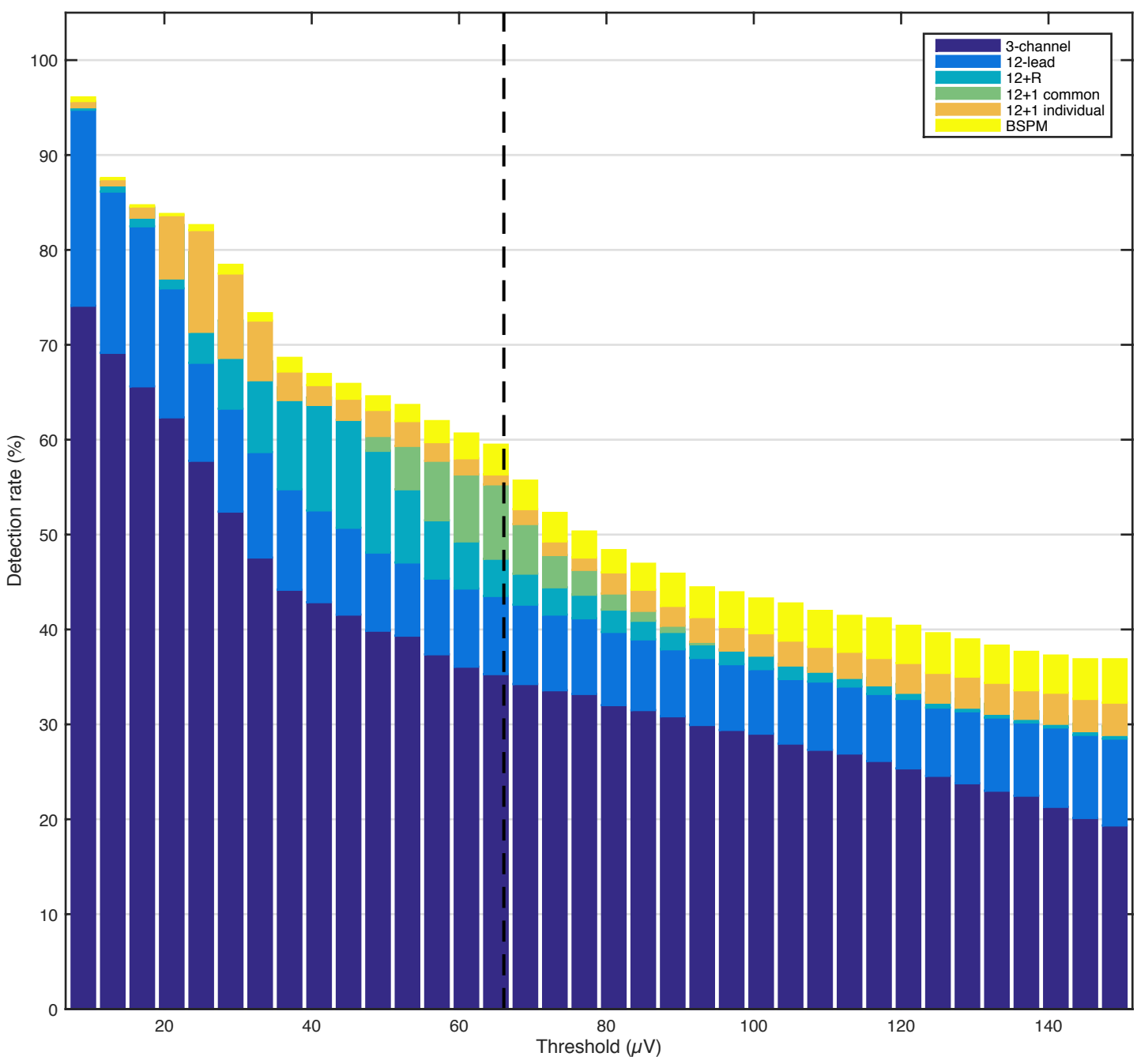

Supplement: Supplementary file 1 — Figure S1. Ischemia detection rates based on K point elevation for different thresholds and ECG lead systems. The dashed line represents the largest K point elevation observed in physiological simulations without any ischemic tissue considering all electrodes. Figure S2. Ischemia detection rates based on K point elevation for single 12-lead channels per AHA segment averaged over the aforementioned set of thresholds. The temporal position of the K point was determined using all 12 leads. [file 530352.f1.zip › threshK_elev.pdf]
